# Children's migration and lifestyle-related chronic disease among older parents ‘left behind’ in india

**DOI:** 10.1016/j.ssmph.2017.03.008

**Published:** 2017-03-31

**Authors:** Jane Falkingham, Min Qin, Athina Vlachantoni, Maria Evandrou

**Affiliations:** Centre for Research on Ageing and ESRC Centre for Population Change, University of Southampton, Southampton, SO17 1BJ, UK

**Keywords:** Migration, Chronic disease, Older parents, Left behind

## Abstract

Lifestyle-related chronic diseases such as cardiovascular diseases and diabetes are now the leading causes of death and disability in India. Interestingly, those Indian states with the highest prevalence of lifestyle-related chronic disease among older adults are also found to have the highest rates of international or internal out-migration. This paper investigates the association between having migrant (adult) children and older parents’ lifestyle-related chronic disease in India. Bi-variate and multivariate analysis are conducted using data from a representative sample of 9507 adults aged 60 and older in seven Indian states from the UNFPA project ‘Building Knowledge Base on Ageing in India’. The results show that for any of the diagnosed conditions of hypertension, diabetes and heart disease, the prevalence among older people with a migrant son is higher than among those without. More specifically, the odds ratio of reporting a lifestyle-related chronic disease is higher among older adults with at least one adult son living in another district, State or outside India than those with their children living closer. This study contributes empirical evidence to the academic and policy debate about the consequences of globalization and urbanization for older people's health status generally, and particularly their risk for reporting chronic diseases that relate to changes in their lifestyle.

## Highlights

• The prevalence of chronic disease is high among ‘left behind’ older parents in India.
• Children's out-migration and (or) migration within different states and (or) different districts are risk factors.
• Selected chronic diseases include diagnosed hypertension, diabetes and heart disease.
• More research is required on the health consequences of globalization and urbanization.

## 1 Introduction

Chronic diseases such as cardiovascular diseases and diabetes are now the leading causes of death and disability in India (Patel et al., 2011, Pappachan, 2011, Diamond, 2011). There is substantial overlap between hypertension, diabetes and heart disease in terms of their aetiology and disease mechanisms; for example, obesity, inflammation, stress and insulin resistance all share common (Cheung & Li, 2012), with research showing that adults with diabetes are more likely than those without diabetes to have hypertension, and are two to four times more likely to have a heart attack, stroke, angina, and coronary artery disease (Nathan et al., 2005, Ali et al., 2010).

The World Health Organization Report on ‘Preventing Chronic Diseases a vital investment’ (WHO, 2005), highlighted the important role of a small set of common, and modifiable, risk factors - including unhealthy diet, physical inactivity and tobacco use - in determining chronic disease, with such life-style choices being influenced in turn by the processes of globalisation and urbanisation. A recent report in India found that older adults in Kerala, Punjab and West Bengal show the highest prevalence of hypertension, diabetes and heart disease (UNFPA, 2012). At the same time, these states witnessed the highest rate of international out-migration (Kerala and Punjab), and a high rate of internal migration (West Bengal) and receipt of remittances (UNESCO & UNICEF, 2012). This raises the intriguing question of whether the increased migration of adult children as a result of globalisation and urbanisation may be playing an intermediate role in the chronic health conditions of their aged parents and if so, whether this opens up areas amenable to policy intervention.

The relationship between adult children's migration and the health of older parents left behind is theoretically unclear, and it is hard to predict whether the migration effects are primarily positive or negative. Previous research suggests that the effect of remittances is likely to change parental health in a positive way. However, little is known about how much of these remittances are received by elderly parents and how this impacts upon health seeking behaviour. Moreover, elderly parents may suffer from emotional distress when their children are absent, and this chronic negative emotion may predispose them to a change in their lifestyle towards less healthy behaviours, such as smoking and consumption of high-fat diets. Also, elderly parents may require physical support (including support in seeking health care) from their adult children, which may be disrupted or unavailable when his/her child migrates.

Previous qualitative studies have found that adult children's migration may yield a benefit or a disadvantage for the health outcome of parents ‘left behind’, depending on the specific country context and the nature of the relationship following the migration of the child(ren) (Miltiades, 2002, Vullnetari and King, 2008, Knodel and Saengtienchai, 2007, Grant et al., 2009). The local culture, the importance of kinship networks, and the social and legal arrangements related to elderly care, are all factors which can intervene in the relationship between adult children's migration and their parents’ health, both positively and negatively. For instance, remittances sent by migrant children may provide funds to facilitate their parents’ access to health services, particularly in countries where free health care or insurance is absent (Biao, 2007). Such remittances may also offer opportunities to older people to improve their diets, leisure or living conditions, and to reduce their experience of psychological distress caused by poverty (Biao, 2007). However, remittances may not always be regular, or indeed directed towards one's elderly parents (Grant et al. 2009). At the same time, children's outmigration has been evidenced to lead to reduced physical and emotional support provided towards one's parents, which can have detrimental effects if there are no substitutes (Qin, 2008). If emotional loss, isolation and a lack of physical care outweigh the gains of out-migration of adult children, such migration may negatively affect their parents’ health. Quantitative studies also reflect mixed findings. For instance, positive effects of children's out-migration on their parents’ health in the origin community have been found in Thailand and Indonesia (Abas et al., 2009, Kuhn et al., 2011). In contrast, negative effects have been found in China and Mexico (Ao et al., 2015, Antman, 2010a, Antman, 2010b). Most of the previous studies have considered older individuals’ self-rated health as the outcome variable, and outcomes linked to lifestyle-related chronic diseases remain relatively under-researched. This paper therefore aims to add to the literature in this field by investigating the association between adult children's migration and older parents’ lifestyle-related chronic disease in India using diagnosed conditions of hypertension, diabetes and heart disease as the outcome variables. To the best of our knowledge this is the first paper to do this.

## 2 Data and methods

### 2.1 Data

This study analyses data collected as part of the UNFPA ‘Building Knowledge Base on Ageing in India (BKPAI)’ project. The BKPAI Survey was conducted in 2011 in seven major demographically advanced states of India - Himachal Pradesh, Punjab, West Bengal, Odisha, Maharashtra, Kerala and Tamil Nadu. Seven states covering the Northern, Southern, Western and Eastern regions of each state were purposively selected, and within each state, a random sampling method was applied to select eligible respondents aged 60 and above. Detailed information about the survey sampling is provided in a previous report (UNFPA, 2012). The BKPAI survey data includes information on older people's mental and physical health, their living arrangements, socio-economic circumstances, including employment status and household assets, as well as information on intergenerational exchanges within the family and participation in social activities. The total sample size interviewed is 9692 individuals. Since the purpose of this paper is to examine the association between adult children's migration and the health of their parents ‘left behind’, 133 childless respondents were excluded, as were respondents with missing data on key variables in the analysis. The final analytical sample used in the paper is 9507 adults aged 60 and above with at least one living child.

Ethical approval analysing these data to study the wellbeing of older people in India has been obtained from the Ethics Committee in the University of Southampton (Ethics ID:21228, permits:13/06/2016).

### 2.2 Measurements

#### 2.2.1 Lifestyle-related chronic disease

The central survey question used in the analysis asks: ‘Has a doctor or nurse ever told you that you have any of the following ailments?’, and has two response options (yes or no). The 20 listed chronic morbidities are: A. Arthritis, rheumatism or Osteoarthritis; B. Cerebral embolism, stroke or Thrombosis; **C. Angina or angina pectoris (heart disease)** (Heart attack, coronary heart disease, angina, congestive heart failure or any other heart problem); **D. Diabetes**; E. Chronic lung disease (emphysema, bronchitis, COPD); F. Asthma (allergic respiratory disease); G. Depression; **H. High blood pressure (hypertension)**; I. Alzheimer's disease; J. Cancer; K. Dementia; L. Liver or gall bladder illness; M. Osteoporosis; N. Renal or Urinary tract infections; O. Cataract; P. Loss of all natural teeth; Q. Accidental injury (in the past one year); R. Injury due to fall (in the past one year); S. Skin disease; and T. Paralysis. In this study, any respondents who reported any of the three following diseases - hypertension, diabetes or heart disease - were defined as having a lifestyle-related chronic disease.

#### 2.2.2 Migrant child (migrant son)

The survey questions ask the place of residence of each child not residing with the respondent, with four response options (within the district; outside the district but within the state; outside the state but within India; and outside India). Also, the survey questions ask the sex and marital status of each child. Having a migrant child is defined in this study as has any child currently living either outside the district or outside the state but within India, or outside India. In the Indian context, dependency in later life is mainly on sons or unmarried daughters, as daughters frequently live elsewhere after marriage. Once married daughters are excluded, very few Indian elders have an unmarried migrant daughter. Thus the analysis focuses on having a migrant son as the key independent variable.

#### 2.2.3 Other control variables

Covariates include a range of factors which have been shown to be important in previous literature exploring factors associated with older people's health outcomes. These include the demographic characteristics of age and sex (Lloyd-Sherlock, Beard, Minicuci, Ebrahim, & Chatterji, 2014) as the impact of risk factors accumulates over individuals’ life course (Ben-Shlomo & Kuh, 2002); socio-economic factors including education, caste, income and household wealth quintile (Mahal et al., 2010, Corsi and Subramanian, 2012, Deepa et al., 2011, Gupta et al., 2012); living arrangements (Samanta, Chen, & Vanneman, 2014); health-risk behaviours such as smoking and alcohol drinking (Laxmaiah et al., 2015); and geographic factors (Kinra et al., 2010, Ramachandran et al., 2004, Joshi et al., 2006, Vijayakumar et al., 2009, Sarkar et al., 2006). Household wealth quintile was computed using Principle Component Analysis based on 30 assets and housing characteristics including: household electrification; drinking water source; type of toilet facility; type of house; type of cooking fuel; house ownership; ownership of a bank or post-office account; ownership of a mattress; a pressure cooker; a chair; a cot/bed; a table; an electric fan; a radio/transistor; a black and white television; a colour television; a sewing machine; a mobile telephone; any landline phone; a computer; internet facility; a refrigerator; a watch or clock; a bicycle, motorcycle or scooter; an animal-drawn cart; a car; a water pump; a thresher and a tractor. This measurement shows a good socio-economic gradient of health outcomes among elder adults in a previous report emanating from the survey (UNFPA, 2012). The number of sons is controlled for in the model, as having more male children may be associated with a higher probability of at least one son migrating out.

### 2.3 Statistical methods

A binary logistic regression model is constructed with ‘lifestyle-related chronic disease’ (recoded as reported hypertension, diabetes or heart disease) as the outcome measure. The first model estimates the bivariate association between having migrant children and reporting a lifestyle-related chronic disease. The second model examines this association after controlling for a range of covariates.

## 3 Results

### 3.1 Descriptive findings

**Fig. 1:**
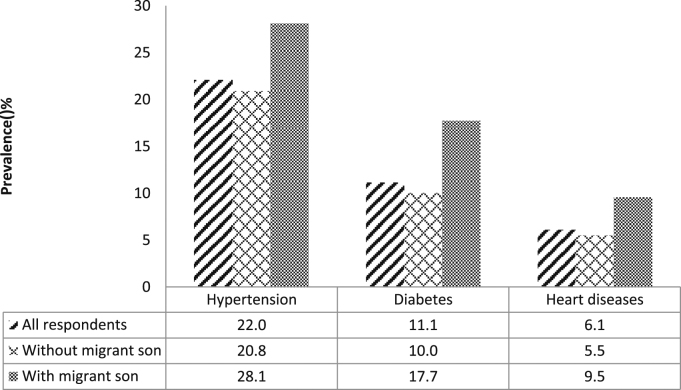
Has at least one migrant son and the prevalence of hypertension, diabetes and heart disease among older adults in India, 2011.

Out of 9507 respondents, 23.9 percent (2269 respondents) reported only one out of three lifestyle-related chronic diseases; 6.4 percent (607 respondents) reported two out of the three chronic diseases; and 0.9 percent (82 respondents) reported all three chronic diseases. Fig. 1 presents the prevalence of hypertension, diabetes and heart disease in three groups: the total study population; among older people with at least one migrant son; and among those without any migrant son, respectively. It shows that for any of the chronic diseases, the prevalence among older people with any migrant son is higher compared to those without a migrant son.

Table 1 presents descriptive statistics for the total analytical sample, that is all individuals aged 60 and above with at least one child in the seven Indian States. The overall prevalence of lifestyle-related chronic disease is 31.1 percent. About 15.2 percent of older adults have at least one son in another district or state or living outside India.^1^ Among this sub-group, the prevalence of lifestyle-related chronic disease is much higher than the average, at 42 percent.

**Table 1:** Distribution of lifestyle-related chronic disease (unweighted).

**Variables**	**Frequency**	**Cases**	**% of lifestyle-related chronic disease**	**P value (Pearson Chi square test)**
**Total**		9,507	31.1	
				
***Has migrant son***				
No	84.8	8061	29.2	.000
Yes	15.2	1446	42.0	
				
***Age group***				
60–69	63.4	6,031	28.8	.000
70–79	26.5	2,520	35.3	
80+	10.1	956	33.9	
				
***Sex***				
Men	47.5	4,591	29.3	.000
Women	52.5	4,988	32.8	
				
***Marital status***				
Widowed	40.3	3,835	32.4	.031
Currently married/living together	59.7	5,672	30.3	
				
***Education***				
None	46.0	4,374	25.0	.000
1–4 years	13.0	1,236	33.7	
5–7 years	13.6	1,290	34.0	
8+ years	27.4	2,607	38.8	
				
***Caste***				
Scheduled Tribe/Scheduled Caste	24.0	2,286	23.0	.000
Other Backward Caste	34.3	3,259	29.8	
Others	39.1	3,718	37.7	
Unknown	2.6	244	25.0	
				
***Income***				
No income	47.6	4,530	31.2	.930
One income source	44.6	4,238	30.9	
Multiple sources	7.8	739	31.5	
				
***Household wealth index***				
Lowest quintile	19.8	1,882	16.5	.000
Second	20.4	1,937	21.8	
Middle	19.8	1,882	31.0	
Fourth	19.9	1,890	36.0	
Highest quintile	20.2	1,916	50.2	
				
***Smoking***				
Never	68.2	6,488	32.3	.000
Ever	31.8	3,019	28.6	
				
***Drinking alcohol***				
Never	92.4	8,789	31.0	.524
Ever	7.6	718	32.2	
				
***No of sons***				
0–1	43.8	4160	28.9	.000
2	34.0	3234	31.1	
3+	22.2	2113	35.4	
				
***Living arrangement***				
Ling alone	6.0	573	23.7	.000
Living with spouse only	14.7	1401	28.1	
Living with children	72.2	6860	31.9	
Others	7.1	673	36.0	
				
***Residence***				
Rural	52.2	4960	27.6	.000
Urban	47.8	4547	35.0	
				
***State***				
Himachal Pradesh	14.9	1417	27.2	.000
Punjab	13.1	1243	42.7	
West Bengal	13.2	1255	34.3	
Odisha	15.3	1452	20.9	
Maharashtra	14.5	1380	21.2	
Kerala	14.1	1336	58.8	
Tamil Nadu	15.0	1424	16.1	

The indicators of socio-economic status appear to have a positive association with the report of lifestyle-related chronic disease. For instance, older people living in households in the highest wealth quintile show a prevalence rate of 50.2 percent compared to 16.5 percent amongst those living in households in the lowest wealth quintile. Older people belonging to Other Castes have a higher prevalence of the disease than those in Scheduled Castes or Scheduled Tribes (37.3 compared to 23 percent), while those with a higher education show a higher prevalence rate than those with lower education.

The level of lifestyle-related chronic disease is higher among older women (32.8 percent) than older men (29.3 percent). One's place of residence seems to play an important role, with elders living in urban areas being more likely to report a lifestyle-related chronic disease than their rural counterparts (35 compared to 27.6 percent). There are also considerable inter-state variations in the prevalence of lifestyle-related chronic disease, as a high level of lifestyle-related chronic disease is found among older adults in Kerala (58.8 percent), Punjab (42.7 percent) and West Bengal (34.3 percent) compared to the other states.

### 3.2 Multivariate analysis results

Table 2 presents the odds ratios for reporting lifestyle related chronic disease among older people. Model 1 shows the bi-variate relationship between has migrant son and the report of a lifestyle-related chronic disease. The odds ratio of 1.76 suggests that older adults who have at least one son in another district or state or outside India are nearly twice as likely to report a lifestyle-related chronic disease than those with children living closer. This effect is attenuated, but still statistically significant, once other control variables are added (Model 2).

**Table 2:** Odds ratio of reporting a lifestyle-related chronic disease.

**Variables**	**Model1**		**Model2**	
	**OR**	**95%CI**	**OR**	**95%CI**
***Has migrant son*** No (ref)				
Yes	1.76***	(1.57–1.98)	1.20**	(1.05–1.37)
				
***Age group*** 60–69 (ref)				
70–79			1.31***	(1.17–1.45)
80+			1.11	(0.93–1.30)
				
***Sex*** Men (ref)				
Women			1.17*	(1.03–1.32)
				
***Marital status*** Widowed (ref)				
Currently married/living together			0.87*	(0.77–0.98)
				
***Education*** None (ref)				
1–4 years			1.15	(0.98–1.34)
5–7 years			1.19*	(1.02–1.38)
8+ years			1.29***	(1.11–1.49)
				
***Caste*** Scheduled Tribe/Scheduled Caste (ref)				
Other Backward Caste			1.12	(0.97–1.29)
Others			1.19*	(1.04–1.36)
Unknown			1.00	(0.72–1.38)
				
***Income*** No income (ref)				
One income source			0.91	(0.82–1.02)
Multiple sources			0.97	(0.80–1.18)
				
***Household wealth index*** Lowest quintile (ref)				
Second			1.30**	(1.09–1.55)
Middle			1.83***	(1.52–2.19)
Fourth			2.19***	(1.81–2.65)
Highest quintile			3.23***	(2.62–3.98)
				
***Smoking*** Never (ref)				
Ever			1.01	(0.90–1.13)
				
***Drinking alcohol*** Never (ref)				
Ever			0.96	(0.79–1.16)
***Number of sons***			1.10***	(1.06–1.15)
				
***Living arrangement*** Living alone (ref)				
Living with spouse only			1.14	(0.87–1.47)
Living with children			0.95	(0.76–1.20)
Others			0.95	(0.72–1.25)
				
***Residence*** Rural (ref)				
Urban			1.03	(0.92–1.14)
				
***State*** Himachal Pradesh (ref)				
Punjab			2.17***	(1.83–2.58)
West Bengal			2.08***	(1.73–2.50)
Odisha			1.11	(0.91–1.34)
Maharashtra			1.00	(0.83–1.21)
Kerala			3.79***	(3.16–4.55)
Tamil Nadu			0.79*	(0.63–0.98)

*** p<0.001

** p<0.01

* p<0.05

Among other important factors, age and gender play a role in the risk of reporting a disease. Women have higher odds than men; and individuals aged between 70–79 have higher odds than other age groups. Socio-economic indicators show an inverse association with lifestyle-related chronic disease, for instance older people with higher education, from wealthier households, or from a higher Caste show higher odds of lifestyle-related chronic disease. There is no difference between urban and rural residence. However, a significant State variation was identified. Older people in Kerala, Punjab and West Bengal show higher odds of reporting a lifestyle-related chronic disease than those in Himachal Pradesh; while those in Tamil Nadu show lower odds. Interestingly, the role of health risk behaviour such as smoking and drinking alcohol is not statistically significant. Finally, older people living with spouse or children show slightly lower odds of reporting lifestyle-related chronic diseases than those living alone; however, this difference is not statistically significant.

## 4 Discussion

The results of this study highlight that out-migration by an adult son could be negatively associated with the health of parents ‘left behind’ in India. Data from representative samples in six of the country's states (Himachal Pradesh, Punjab, West Bengal, Odisha, Maharashtra, Kerala and Tamil Nadu) show a higher risk of lifestyle-related chronic disease for older adults with children who have migrated overseas or to another state or district in India. Building on existing research, the original contribution of this paper lies in shifting away from self-rated health as the outcome indicator, in order to consider specific chronic diseases which are related to changes in older people's lifestyles, namely hypertension, diabetes and heart disease. By grouping the three chronic diseases in the outcome variable, the study could avoid, to some extent, an underestimation in their prevalence as a result of the self-reported nature of the measures (Vellakkal et al., 2013).

### 4.1 Higher chronic disease risk for elderly people with a migrant son

Using self-reported health outcomes, this analysis found a significant association between having at least one migrant son and their older parents reporting a lifestyle-related chronic disease. After controlling for a range of demographic, socio-economic, risk behaviour and geographic factors, the association persists. One possible explanation of this association may relate to existing research which shows that parents with migrant children may suffer from chronic stress, a change in their lifestyle towards less healthy behaviours, and a lower likelihood of using health care (Pickering, 1999, Antman, 2010a, Antman, 2010b). In the first instance, the stress resulting from children's out-migration may have direct effects on the disease processes. Several qualitative studies have found that in transitional societies including India, out-migration of adult children is often accompanied by increased loneliness and isolation, which are difficult to address through mechanisms of formal care (Miltiades, 2002, Vullnetari and King, 2008, Grant et al., 2009). Feelings of loneliness and isolation may in turn lead to constant worrying, anxiety, pessimism and depression among older parents, which can compromise one's health in the long run. Indeed, studies have suggested that chronic exposure to stress may have an influence on increased blood pressure, elevated blood glucose levels and an increased risk for heart disease, which is mediated through the symptomatic nervous system, the hypothalamic–pituitary–adrenal axis, and the renin–angiotensin–aldosterone system (Surwit and Schneider, 1992, Gasperin et al., 2009). In addition, individuals under stress might try to relieve their tension by consuming more food, tobacco and alcohol, which is harmful to one's health (Epel, Lapidus, McEwen, & Brownell, 2001).

A second mechanism which could explain the association between having migrant children and reporting lifestyle-related chronic diseases could operate through the receipt of remittances from migrant children, which is likely to change the lifestyle and behaviour patterns of their parents. In the past few decades, many Indian states such as Kerala and Punjab have witnessed a substantial proportion of the households’ cash income being attributed to migrant earnings (Haberfeld et al., 1999, Rogaly et al., 2001, Mosse et al., 2002). Evidence shows that such increases in household incomes are contributing to diets which are high in energy, fats, salt and sugar, and which are negatively associated with health outcomes (Basu et al., 2002, Yoshida et al., 2007). As a result, changes in individuals’ lifestyle have been cited as a contributing cause of the increasing prevalence of lifestyle-related diseases in India (Pappachan, 2011). A third explanation of this paper's key finding relates to the issue of access to, and utilization of, health care among older people. Previous research has found that elderly individuals with migrant children are less likely to receive intra-household elderly care and to be taken to the hospital compared to older people whose adult children live in closer proximity (Qin, 2008). In the UNFPA dataset, that only those respondents who report suffering from ill health in the previous 15 days are asked about seeking treatment and so it was not possible to explore this aspect in the multivariate model. To shed some light on this, we have however separately analysed the association between having a migrant son and treatment seeking for each of the twenty ailments listed in the survey. The results are included in the Supplementary materials (Table A1) and show that among older people reporting suffering from a cerebral embolism, stroke, cancer or loss of all natural teeth, those with a migrant son are more likely not to have sought treatment for this condition in the last three months than those without a migrant son; however the sample sizes in each case are small. Nevertheless the results are supportive of the hypothesis that those older people with a migrant son may be less likely to seek care. The overall finding of a negative association between migrant child and parental health is consistent with previous studies in Mexico, Thailand and China (Antman, 2010a, Antman, 2010b, Adhikari et al., 2011, Ao et al., 2015).

This study also found a significant socio-economic status (SES) gradient in terms of the report of lifestyle-related diseases, particularly in terms of household wealth, education and Caste, suggesting that individuals with a higher SES are at an increased risk of reporting lifestyle-related diseases when compared to those lower on the socioeconomic distribution. These findings are consistent with other studies in India focusing on self-reported health outcomes (Mahal et al., 2010, Corsi and Subramanian, 2012), and using measurable indicators (Lloyd-Sherlock et al., 2014). Notwithstanding this finding, it should be noted that over the past decade, the prevalence of diabetes and cardio-metabolic risk factors have rapidly increased among lower SES groups in India as well (Deepa et al., 2011, Gupta et al., 2012).

### 4.2 Limitations

There are certain limitations in this study which ought to be taken into account when interpreting the results. Firstly, given the cross-sectional design of the BKPAI survey, it is not possible for this study to ascertain causality between having migrant adult children and their parents’ health outcomes in the origin community, allowing only for an identification of statistically significant associations between the two. However, a prior we might expect the parents of migrants to report better health outcomes than the parents of non-migrants, assuming the healthy selection of migrants and parent-child health correlation due to genetic or environmental factors (Giles & Um, 2007), whilst in fact the converse is found. Secondly, no information is available on the older parents’ emotional status and possible stress following their adult children's migration, or on the parents’ food and other types of consumption, and healthcare utilization, all of which would facilitate the exploration of alternative mechanisms. Future research would benefit from longitudinal data and more information about intermediate risk factors in the association between having migrant children and reporting chronic diseases, such as abnormal blood pressure, glucose levels, blood lipids, and body weight (WHO, 2005).

## 5 Conclusions and policy implications

India has set up specific national targets and indicators aimed at reducing the number of global premature deaths from non-communicable diseases by 25 percent by 2025 (WHO, 2015). This study contributes empirical evidence to the academic and policy debate about the possible consequences of globalization and urbanization for older people's health status generally, and particularly their risk for reporting chronic diseases which relate to changes in their lifestyle. International migration and internal migration within different states or districts is likely to continue being a key feature of economic and social life in India (UNESCO & UNICEF, 2012), while population ageing will continue contributing policy challenges at both the individual and household levels (Government of India, 2011). Against this background, balancing economic growth with the maintenance and improvement in population health will be important in order to ameliorate any negative impacts upon older people's wellbeing. Maintaining older adults’ contact with their migrant children, being visited by children living in close proximity, and involving older people in a range of social activities, have been shown to comprise effective coping strategies for older adults to overcome any negative consequences of their children's migration (Ashfaq, Abbasi, Ali, & Habiba, 2016). This study further underlines the importance of policy makers paying attention to the migration status of adult children and, in the Indian context, particularly migrant sons, and the potential role of social networks in maintaining good health in later life.

## Conflict of interest statement

There is not conflict of interest.

## Footnotes

1 8.5% of respondents have at least one son outside the district; 3.9% have at least one son living outside the state; and 3.7% have at least one son living outside the country.
