# Supplementary material for: Children's migration and lifestyle-related chronic disease among older parents ‘left behind’ in india
Source: SSM Popul Health. 2017 Mar 31;3:352–7. doi: 10.1016/j.ssmph.2017.03.008 (PMC5769047; doi:10.1016/j.ssmph.2017.03.008)
Supplement: Supplementary file 1 — Supplementary material [file mmc1.docx]

Appendix

Table A1 Percentage of older persons (aged 60+) who report a selected chronic ailment during last 3 months not seeking treatment for that aliment, according to the migration status of child(ren)

| Chronic conditions | without migrant son | with migrant son |  | Cases |
| --- | --- | --- | --- | --- |
| Arthritis | 24.2 | 23.6 |  | 648 |
| Cerebral embolism, stroke or Thrombosis | 14.7 | 36.4 | * | 19 |
| Angina or angina pectoris | 13.0 | 10.9 |  | 73 |
| Diabetes | 8.0 | 7.8 |  | 84 |
| Chronic lung disease | 23.4 | 37.5 |  | 35 |
| Asthma | 18.8 | 14.2 |  | 124 |
| Depression | 65.3 | 51.9 |  | 95 |
| High blood pressure | 9.3 | 10.8 |  | 200 |
| Alzheimer’s disease | 57.4 | 70.0 |  | 65 |
| Cancer | 19.4 | 100.0 | ** | 10 |
| Dementia | 73.1 | 68.8 |  | 60 |
| Liver or gall bladder illness | 30.6 | 52.4 | * | 55 |
| Osteoporosis | 46.7 | 31.3 |  | 94 |
| Renal or Urinary tract infections | 41.9 | 50.0 |  | 106 |
| Cataract | 53.1 | 59.2 |  | 623 |
| Loss of all natural teeth | 78.5 | 87.2 | ** | 860 |
| Accidental injury (in past one year) | 36.4 | 43.8 |  | 43 |
| Injury due to fall (in the past one ye | 42.3 | 49.1 |  | 141 |
| Skin disease | 43.2 | 42.9 |  | 97 |
| Paralysis | 15.4 | 19.0 |  | 27 |

**p<0.01

*p<0.05
